# Supplementary material for: ProbeTools: designing hybridization probes for targeted genomic sequencing of diverse and hypervariable viral taxa
Source: BMC Genomics. 2022 Aug 12;23:579. doi: 10.1186/s12864-022-08790-4 (PMC9371634; doi:10.1186/s12864-022-08790-4)
Supplement: Supplementary file 1 — Additional file 1. [file 12864_2022_8790_MOESM1_ESM.pdf]

HA

Significantly enriched

Targeted by panel	False	True	Total
False	402 (1.0%)	4,291 (10.5%)	4,693 (11.5%)
True	1,195 (2.9%)	34,957 (85.6%)	36,152 (88.5%)
Total	1,597 (3.9%)	39,248 (96.1%)	40,845 (100.0%)

NA

Significantly enriched

Targeted by panel	False	True	Total
False	125 (0.4%)	2,539 (7.5%)	2,664 (7.8%)
True	916 (2.7%)	30,496 (89.5%)	31,412 (92.2%)
Total	1,041 (3.1%)	33,035 (96.9%)	34,076 (100.0%)

M

Significantly enriched

Targeted by panel	False	True	Total
False	234 (1.0%)	848 (3.5%)	1,082 (4.4%)
True	210 (0.9%)	23,245 (94.7%)	23,455 (95.6%)
Total	444 (1.8%)	24,093 (98.2%)	24,537 (100.0%)
