# Supplementary material for: ProbeTools: designing hybridization probes for targeted genomic sequencing of diverse and hypervariable viral taxa
Source: BMC Genomics. 2022 Aug 12;23:579. doi: 10.1186/s12864-022-08790-4 (PMC9371634; doi:10.1186/s12864-022-08790-4)
Supplement: Supplementary file 2 — Additional file 2. [file 12864_2022_8790_MOESM2_ESM.pdf]

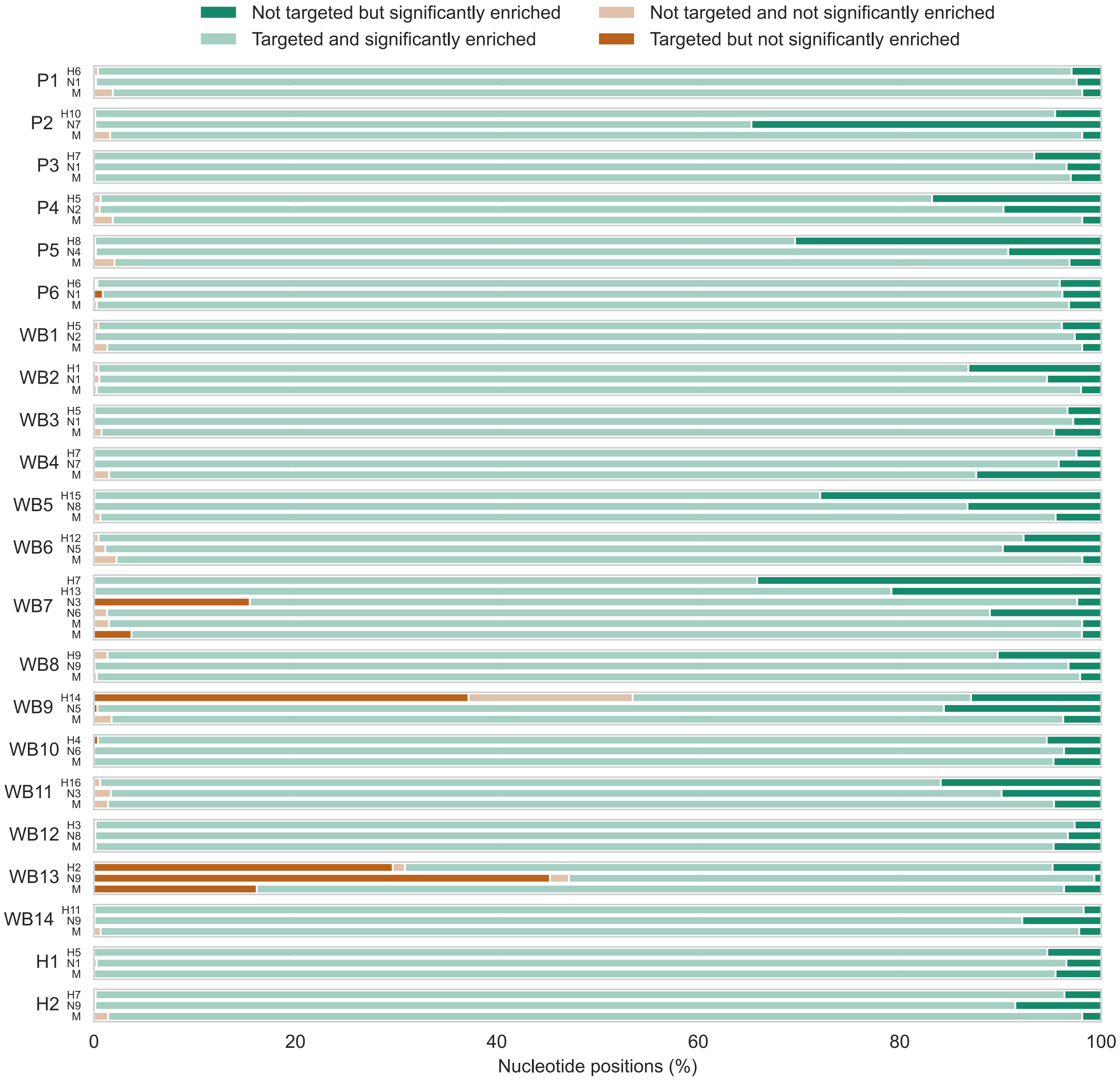

Not targeted but significantly enriched
Not targeted and not significantly enriched
Targeted and significantly enriched
Targeted but not significantly enriched
P1
H6
N1
M
P2
H10
N7
M
P3
H7
N1
M
P4
H5
N2
M
P5
H8
N4
M
P6
H6
N1
M
WB1
H5
N2
M
WB2
H1
N1
M
WB3
H5
N1
M
WB4
H7
N7
M
WB5
H15
N8
M
WB6
H12
N5
M
WB7
H7
H13
N3
N6
M
M
WB8
H9
N9
M
WB9
H14
N5
M
WB10
H4
N6
M
WB11
H16
N3
M
WB12
H3
N8
M
WB13
H2
N9
M
WB14
H11
N9
M
H1
H5
N1
M
H2
H7
N9
M
0
20
40
60
80
100
Nucleotide positions (%)
